# A Case in Practice

**Published:** 1884-10

**Authors:** C. H. Eccleston

**Affiliations:** Oxford, N. Y.


### A CASE IN PRACTICE.

BY DR. C. H. ECCLESTON, OXFORD, N. Y.

Some three months ago a young lady, aged about seventeen years, healthy appearance, but nervous temperament, called to consult me in regard to her superior central incisors. I found upon examination that nearly all of the front teeth, upper and under, were affected with *atrophy*, which gave the teeth a shriveled appearance on the cutting edge and about one-third of the crown, the superior centrals being the most affected, as about one-third of the crowns were gone, as though cut off with cutting pliers while in a soft state. They presented no decay of consequence, but had irregular, discolored grooves and numerous indentations on what would be the cutting edge, but none on the remaining portion of the crowns. To make the case more complicated, the left central overlapped the right. The first step taken to restore the defective centrals was to file the cutting edges down to a surface and marginal edge with the remainder of the crowns. This done, an impression was taken to obtain a model to which porcelain points could be made. The porcelain points were not made solid, like ordinary tooth points, but hollow and slightly undercut (rather delicate work), to retain the cement used to attach them to the natural crowns. To attach the porcelain points I prepared the natural crown in about the manner I would for building down the points with gold, viz: burring in a cavity across the whole width of the crown, about one thirty-second of an inch deep, with a slight undercut, when I adjusted two gold retaining screws for each crown, one on each side of the nerve center, allowing the screws to project enough to reach nearly to the bottom of the hollow porcelain points. In this case I used phosphate filling to attach the porcelain points, in about the same manner as in ordinary pivot tooth setting. As to the durability of such work as compared with building down with gold, I am unable to say; it certainly looks much better, and is not noticeable except to a critical eye.

We have casts of this case, and they indicate a very neat and successful operation. Dr. Eccleston, a practical tooth manufacturer, carved new tips to exactly restore the lost contour of the teeth. They were attached as described, and thus the unsightliness of gold restoration was avoided. Those who desire to do such work and have not the conveniences for manufacturing any desired restorative in porcelain, could have that part of the work done by Dr. Eccleston. He has been our *dernier ressort* for nearly twenty years.—EDITOR.

